# Whole-genome resequencing identified QTLs, candidate genes and Kompetitive Allele-Specific PCR markers associated with the large fruit of Atlantic Giant (*Cucurbita maxima*)

**DOI:** 10.3389/fpls.2022.942004

**Published:** 2022-07-22

**Authors:** Liu Pan, Min Wang, Yating Yang, Chen Chen, Haibo Dai, Zhiping Zhang, Bing Hua, Minmin Miao

**Affiliations:** ^1^College of Horticulture and Plant Protection, Yangzhou University, Yangzhou, China; ^2^Joint International Research Laboratory of Agriculture and Agri-Product Safety of Ministry of Education of China, Yangzhou University, Yangzhou, China; ^3^Key Laboratory of Plant Functional Genomics of the Ministry of Education/Jiangsu Key Laboratory of Crop Genomics and Molecular Breeding, Yangzhou University, Yangzhou, China

**Keywords:** Atlantic Giant, fruit size, genome-wide resequencing, KASP marker, *Cucurbita maxima*

## Abstract

Atlantic Giant (AG) pumpkin (*Cucurbita maxima*) produces the world’s largest fruit. Elucidating the molecular mechanism of AG fruit formation is of scientific and practical importance. In this research, genome-wide resequencing of an F_2_ population produced by a cross between AG and its small-fruit ancestor Hubbard was used to identify quantitative trait loci (QTLs) and candidate genes. Transgressive segregation of fruit size-related traits was observed in the F_2_ population, suggesting that fruit size was a quantitative trait controlled by multiple genes. A genetic map with an average physical distance of 154 kb per marker was constructed, and 13 QTLs related to fruit size were identified using bin-map construction. RNA sequencing analysis revealed that pathways associated with assimilate accumulation into the fruit, including carbohydrate metabolism, were significantly enriched in differentially expressed genes. According to the predicted impact of mutation on the biological function of certain proteins, 13 genes were selected as candidate genes associated with fruit size, among which two phytohormone-related genes, *CmaCh17G011340* (a flavin-containing monooxygenase) and *CmaCh04G029660* (a leucine-rich repeat protein kinase) were chosen for further investigation. Finally, one insertion-deletion (inDel) and three single nucleotide polymorphisms (SNPs) were successfully transformed to Kompetitive Allele-Specific PCR (KASP) markers. The novel QTLs and candidate genes identified provide insights into the genetic mechanism of large fruit formation of AG, and the genetic map and tightly linked KASP markers developed in this study can be employed for marker-assisted breeding to alter fruit size of *C. maxima*.

## Introduction

The world’s largest fruit is produced by the Atlantic Giant (AG) pumpkin (Pan et al., 2021). Each year, for giant pumpkin growers, giant pumpkin weigh off is the last of the great outdoor festivals. Due to the efforts of these growers from both aspects of breeding and cultivation, new records are created for the weight of the largest pumpkin every 1–2 years. To date, the world record of the fruit weight of AG is 1226 kg (Guinness World Records, 2021). It is believed that the current cultivated AG originated from the Hubbard squash (Sanjur et al., 2002), and the dramatic increase in the fruit weight of the modern AG from its ancestor has occurred only in the last 100 years (Hu et al., 2011).

It is interesting and important to know the genetic and physiological changes between AG and Hubbard caused by artificial selection and crossing during the last century. To date, only several studies have focused on the differences in the morphology and physiology between the two varieties. Basically, on the source side, the leaf area difference between the two types of pumpkins is slight (Savage et al., 2015; Pan et al., 2021). The leaf photosynthetic rate (Pn) of the two varieties was not significantly different at anthesis stage (Savage et al., 2015). However, at the fruit fast-growing stage, the Pn of the fruit-carrying leaf was higher in AG than in Hubbard (Pan et al., 2021). Along the assimilate translocation pathway, the intersecting surface of both the xylem and the phloem in both the pedicels and petioles of Hubbard was smaller than that of AG, while the phloem flow rate was similar between the two pumpkins (Savage et al., 2015; Pan et al., 2021). On the sink side, data from both Nakata et al. (2012) and our study (Pan et al., 2021) indicated that the larger size of AG fruit is due to faster increases in both the number and size of the fruit cells. Logically, the abovementioned differences should be governed by genetic divergency between the two varieties. However, unfortunately, which genetic loci have been altered by breeding and selection from Hubbard to AG during the last century remains largely unclear. The only certainty is that polyploidy is not the reason for the large fruit formation of AG (Tatum et al., 2006; Nakata et al., 2012).

Fruit size is an important quality and yield trait of cucurbits and other fruiting crops. As a result, the genetic mechanism of fruit size has been widely investigated in different plants using different genetic tools. Basically, fruit size is determined by the number and size of fruit cells and derived by cell proliferation and cell expansion during organ growth. A number of key components controlling cell proliferation have been identified in different plants. In maize (*Zea mays*), *cellulose synthase-like D1* promotes cell division by participating in cell wall polysaccharide formation (Li N. et al., 2018). In legumes, a KIX domain protein gene *bigger organs* and an ortholog of *PEAPOD* gene *ELE1* interact with each other and negatively regulate organ size by repressing cell proliferation (Li et al., 2019). The cauliflower (*Brassica oleracea*) curd development-associated gene (*CDAG1*) can lead to increased cell number and enlarged organ size (Li et al., 2017). In Arabidopsis (*Arabidopsis thaliana*), *AINTEGUMENTA* and *ErbB-3* binding protein 1 (*ZmEBP1*) can increase organ size by accelerating cell division (Wang Q. L. et al., 2016; Wang T. Y. et al., 2016; Ding et al., 2018). In Arabidopsis, soybean (*Glycine max*) and cucumber (*Cucumis sativus*), STERILE APETALA family genes also promote cell proliferation by modulating PPD or BIG SEEDS1 (Li W. Y. et al., 2018; Yang et al., 2018; Yin et al., 2020). Genes associated with cell expansion also play key roles in controlling organ size. Wang Q. L. et al. (2016) reported that the maize ADP-ribosylation factor *ZmArf2* promotes cell expansion and enhances organ size in Arabidopsis. In addition, some uncharacterized genes, such as *Cell Size Regulator* in tomato (*Solanum pimpinellifolium*) and *DUF4* in Arabidopsis, were reported to promote organ size by increasing cell size rather than cell number (Mu et al., 2017; Chen et al., 2018). *Cytochrome P450* genes have been found to be involved in controlling organ size and development in Arabidopsis, rice, soybean, tomato (*Solanum lycopersicum*) and sweet cherry (*Prunus avium*; Chakrabarti et al., 2013; Yang et al., 2013; Zhao et al., 2016; Nobusawa et al., 2021). Phytohormones are important factors promoting cell division and growth. It has been reported that auxin, cytokinin, gibberellin, brassinosteroid (BR) and genes (Davies, 1987; Mandava, 1988; Mok and Mok, 1994; De León et al., 2004; Achard et al., 2009; Devoghalaere et al., 2012; Jiang et al., 2012; Qi et al., 2017) in their signaling pathways are closely related to organ size. In addition, polyploidy and locule number are associated with the fruit size of some species (Cheniclet et al., 2005; Muńos et al., 2011). Pan et al. (2020) surveyed several cucurbit genomes and identified more than 200 homologs of organ size-related genes, including *cytochrome P450*, *CSR* (cell size regulator) and *CNR* (cell number regulator).

The genomics research and genome sequencing of *Cucurbita maxima* have achieved great advances in recent years (Sun et al., 2017). Several high-density genetic maps were constructed using simple sequence repeat (SSR) markers or single nucleotide polymorphism (SNP), and quantitative trait loci (QTLs) of interested agronomic traits, such as dwarf vine, seed shape and weight, fruit carotenoid content and other fruit-associated traits were identified (Van Der Knaap et al., 2014; Zhang et al., 2015; Kaźmińska et al., 2018, 2020). These studies provide useful information and tools to unlock the mystery of the formation of world’s largest fruit. In this research, a population with 102 F_2_ individuals was generated from a cross between AG and Hubbard. The population was then resequenced, and a high-density genetic map was developed. Afterward, QTLs for fruit cell number, fruit cell volume, fruit weight and fruit volume were identified, and 4 Kompetitive Allele-Specific PCR (KASP) markers were established. These studies will facilitate the identification of key genes regulating fruit size. In addition, the high-density genetic map and linked markers will provide useful tools for marker-assisted selection (MAS) in *C. maxima* breeding programs.

## Materials and methods

### Plant materials and phenotyping

The seeds of two parents of *C. maxima*, Hubbard and Atlantic Giant (AG) were obtained from the Sustainable Seed Company (Covelo, California, United States). A segregating population of 102 F_2_ plants was constructed from a cross between the female parent AG and the male parent Hubbard. Self-pollination was further performed for these F_2_ individuals to generated F_2:3_ families. Two parents, F_1_ and F_2:3_ plants were grown in the spring of 2020 in plastic tunnels at Yangzhou University (119°26′E, 32°24′N), Yangzhou, China. Plant management was performed according to Nakata et al. (2012) with a few modifications. Basically, the row and plant spacing were 2 and 5 m, respectively. A hole 1 m in diameter and 50 cm deep was dug for every plant. Fertilizers, including 20 kg of cow manure, 10 kg of rapeseed lees, 10 kg of CaSO_4_ fertilizer and 10 kg of fertilizer (N:P_2_O_5_:K_2_O = 15:10:15), were applied to each plant. Half of the fertilizers were put into the hole, and the rest were spread over the field. Only one fruit remained per plant (usually between the 14th and 18th nodes from bottom to top of the plant). Water and fertilizer management was performed to ensure the consistency of the growth condition among all plants.

A randomized block design was performed to collected the phenotypic data. Basically, twenty individuals were planted for each line (two parents, the F_1_ population, 102 F_2:3_ families and six cultivars with distinct fruit sizes) as a plot (200 m^2^) and the experiment was repeated 3 times in different plastic tunnels. The weight (W1), volume (V1), cell volume (CV) and the number of sarcocarp cells (CN) of the fruit was measured according to our previous study (Pan et al., 2021). The weight (W2) and volume (V2) of the sarcocarp were obtained after pumpkin fruit was cut in half and the seeds and placenta were removed. Each phenotypic value was the mean of 3 replicates and the data of each replicate were calculated from 10 randomized selected plants in the plots. Correlations were calculated using the Pearson correlation coefficient by Statistica 12 software (Statsoft Inc., United States) at *p* ≤ *0.05.*

### Nucleic acid isolation and sequencing

Genomic DNA of the two parents and all 102 F_2_ plants was extracted from young leaves using a Plant Genomic DNA Extraction Kit (TIANGEN, Beijing, China) according to the manufacturer’s instructions.^1^ RNA was extracted from 20 days after anthesis (the exponential growth period) fruit sarcocarp tissues using RNAiso Plus (Takara, Dalian, China) according to the manufacturer’s instructions.^2^ For RNA extraction, each sample was a mix of nine parts from three fruits, and three independent biological replicates were performed (Pan et al., 2021). The purity and integrity of the RNA and DNA samples were determined by 1% agarose gel electrophoresis, and the concentration of nucleic acids was measured in a NanoDrop. High-throughput sequencing for DNA and RNA libraries was performed by BGI Tech^3^ using a BGISEQ-500/MGISEQ-2000 system or Novogene^4^ using an Illumina HiSeq TM 4000 system, respectively.

### Data analysis and single nucleotide polymorphism genotyping

To obtain clean reads, the adapter sequences, low-quality reads (Q ≤ 10) and N > 5% reads were filtered out by the Soapnuke tool (1.5.6, BGI Tech). Then, using Burrows–Wheeler Aligner (BWA) software, all clean reads were mapped with the *C. maxima* genome sequence^5^ (Li and Durbin, 2009). Picard tools^6^ were used for the realignment of inDel regions and removal of PCR duplicates. Genome analysis toolkit (GATK) software was employed to call and filter genome-wide SNPs with default parameters (McKenna et al., 2010). The heterozygous alleles in both parents were removed during the process.

### Bin-map construction and quantitative trait loci analysis

SNPs on the same scaffold with identical genotypes across the F_2_ population were concatenated into a bin using SNPbinner (Chen et al., 2014). Bins less than 5 kb were initially filtered out, and bins with an extreme segregation distortion (*P* < *0.001*) by the χ^2^ test were eliminated. The filtered bin-markers were then employed for map construction by LepMAP3 (Rastas, 2017). Linkage groups (LGs) were constructed using likelihood odds (LOD) ratios ≥ 5.0 and genetic map distances were calculated with the Kosambi mapping function (Kosambi, 1944). R/qtl software was employed for QTL identification based on the inclusive composite interval mapping (ICIM) model (Broman et al., 2003). Calculations using 1000 permutations were used to set the significant LOD threshold of QTLs. QTLs of the six traits associated with fruit size, CN, CV, W1, V1, W2 and V2 mentioned above, were identified.

### Bioinformatics analysis of RNA sequencing data

Raw reads were filtered to eliminate adaptor sequences, low-quality reads with > 10% unknown nucleotides and poly-N. The clean reads were mapped to the *C. maxima* genome (see text footnote 5) using hierarchical indexing for spliced alignment of transcripts 2 (HISAT2; Kim et al., 2019). Read counts of annotated genes were calculated using StringTie (Pertea et al., 2016). fragments per transcript kilobase per million fragments mapped (FPKM) was used to estimate gene expression levels (Trapnell and Salzberg, 2009). Genes meeting the log2 (fold change) ≥ 1 and false discovery rate (FDR) ≤ 0.01 were defined as differentially expressed genes (DEGs) between the two parents. The Kyoto Encyclopedia of Genes and Genomes (KEGG) pathway enrichment was analyzed according to biological pathways.^7^

### qRT–PCR for candidate genes

The expression of genes located in the QTL regions that were associated with more than one trait and differentially expressed between the two parents was further verified by Quantitative real-time polymerase chain reaction (qRT–PCR). The experiments were conducted using the same RNA samples as those used for RNA sequencing mentioned above. The primers for this study are listed in Supplementary Table 1.

### Evaluation of biological function of single nucleotide polymorphisms

The impact of the “missense_variant” SNPs on the biological function of certain proteins was evaluated using PROVEAN (Choi et al., 2012), and only SNPs with a prediction of “Deleterious” calculated in alternative directions were selected.

### Development and analysis of Kompetitive Allele-Specific PCR markers

Five missense SNPs in key candidate genes and inDels in the coding region of genes located in QTL regions were selected to develop KASP markers. Two allele-specific forward primers and one common reverse primer (Supplementary Table 2) for KASP™ assays were designed. The developed KASP markers were used to differentiate the polymorphism by genotyping the two parents, the entire F_2_ population and several other *C. maxima* cultivars with distinct fruit sizes.

### *In silico* analysis

Sequences of Flavin-containing monooxygenase family protein (YUCCA) and leucine-rich repeat receptor-like protein kinase (LRR-RLK) proteins were collected from (see text footnote 5), TAIR,^8^ and previous studies (Abu-Zaitoon, 2014; Song et al., 2015). Multiple sequence alignments were carried out using DNAMAN 6.0 (LynnonBiosoft, United States), and phylogenetic relationships were established by neighbor joining using aligned amino acid sequences analyzed by Mega X (Institute of Molecular Evolutionary Genetics, The Pennsylvania State University, University Park, United States).

## Results

### Valuation of fruit size-associated traits

**FIGURE 1:**
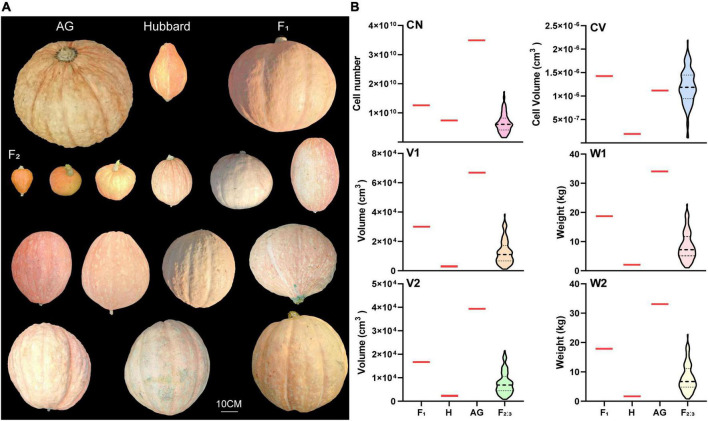
Fruit phenotypic characterization of two parents “Atlantic Giant” and “Hubbard”, the F_1_ hybrid and the F_2:3_ groups. **(A)** Fruit morphology. **(B)** Violin plots of cell number (CN), cell volume (CV), fruit volume (V1), fruit weight (W1), fruit volume without seeds and placenta (V2) and fruit weight without seeds and placenta (W2) of Atlantic Giant (AG), Hubbard (H), F_1_ and F_2_ group. Black horizontal lines display the median and black dotted lines display 25th and 75th percentiles.

Although the fruit weight of AG in this study was much lighter than the world heaviest fruit record (1226 kg) due to the limited growth condition, several fruit-size associated traits, including V1, W1, V2 and W2 of AG, were still much higher than those of its small fruit ancestor Hubbard. All F_1_ plants showed an intermediate phenotype in these traits. Transgressive segregation was found in the F_2:3_ population for all investigated traits, and the average value was skewed toward the small fruit-size parent (Figure 1). The correlation analysis indicated that the six tested traits were closely correlated, except for the correlation between CN and CV (Supplementary Table 3).

### Sequencing, genotyping, and genetic map construction

**FIGURE 2:**
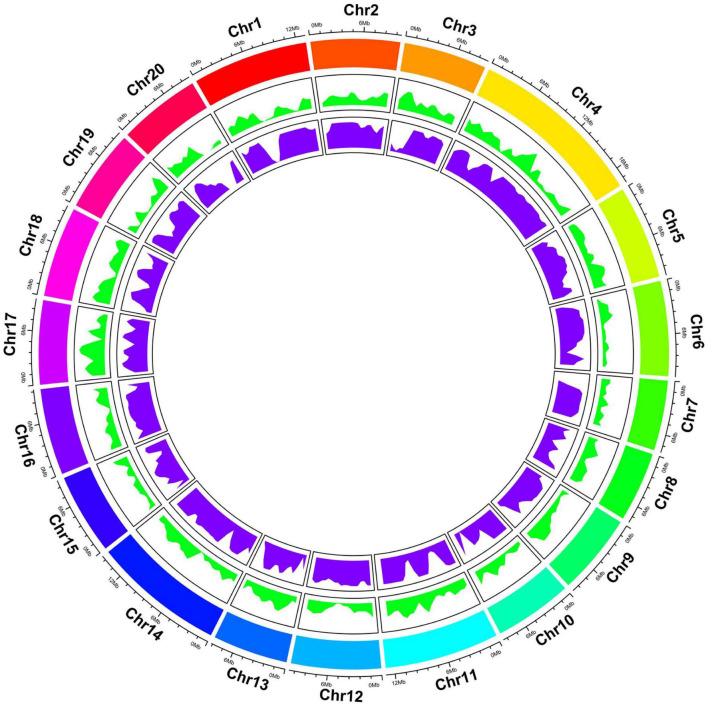
Overview of the Genome-wide resequencing among two parents (AG and Hubbard) and 102 F_2_ plants. Tracks from outside to inside: Chromosome numbers and lengths, Single Nucleotide Polymorphisms (SNPs) and Bins.

Genome-wide resequencing (GWRS) was performed for two parents (AG and Hubbard) and 102 F_2_ plants. As shown in Supplementary Table 4, approximately 44.32 and 44.52 million cleaned reads from parents Hubbard and AG, respectively, and an average of 4.23 million cleaned reads from F_2_ individuals were obtained. Among these reads, the average GC content was 38.56% and the Q30 score was more than 88.41%. The average depths of Hubbard, AG and F_2_ plants were 7, 7 and 1, respectively. Of these reads, 76.94% from AG, 74.34% from Hubbard and 78.87% from F_2_ plants were mapped to the genome of *C. maxima* and employed for SNP calling. Finally, a total of 208626 polymorphic SNPs between the two parents were obtained. The SNPs were found to be distributed across all 20 LGs, as illustrated in Figure 2. The detailed information of these SNPs is listed in Supplementary Table 5. Therefore, these SNPs were further combined into 2973 bin markers (Qi et al., 2021). These bin markers were used to construct the 20 LGs, and individual LGs consisted of 95 to 284 markers for LG7 and LG4, respectively, with an average of 148.25 markers per LG. The size of the LGs ranged from 69.63 to 189.56 cM for LG7 and LG4, respectively. The genetic map spans a total of 2337.88 cM, and the mean interval between bin markers was approximately 0.96 cM, and the maximum distance between the bins ranged from 4.42 to 18.44 cM. According to the pumpkin genome size (373.9 Mb), the map had an mean physical spacing of 154 kb per bin (Table 1). The recombination bin-map, heatmaps and synteny analysis are shown in Supplementary Figures 1–3, and the obtained results indicated that a genetic map with high quality and good collinearity was constructed.

**TABLE 1:** Basic information statistics of the genetic map.

LG ID	Spearman (*R*^2^)	Total distance (cM)	Total bin marker	Unique bin marker	Mean distance (cM)	Max Gap (cM)	Gap < 5 cM (%)
LG01	0.999872983	150.27	170	148	1.02	10.44	98.65
LG02	0.999959086	121.221	170	151	0.8	8.92	98.68
LG03	0.999888817	130.137	155	134	0.97	15.15	98.51
LG04	0.997327309	189.561	284	216	0.88	12.51	99.54
LG05	0.999454392	114.41	160	128	0.89	8.92	96.88
LG06	0.999896691	109.936	132	108	1.02	8.41	98.15
LG07	0.999839022	69.633	95	87	0.8	4.42	100
LG08	0.938374327	133.1	132	94	1.42	18.44	94.68
LG09	0.999131348	103.992	129	108	0.96	4.92	100
LG10	0.999923118	93.901	109	92	1.02	11.99	97.83
LG11	0.999992659	128.076	187	175	0.73	9.42	99.43
LG12	0.999702745	110.412	173	140	0.79	8.92	99.29
LG13	0.999953664	99.808	110	98	1.02	11.47	96.94
LG14	0.9999501	139.965	181	155	0.9	10.44	98.71
LG15	0.99998174	106.182	122	109	0.97	10.44	96.33
LG16	0.999962663	97.211	141	111	0.88	9.42	99.1
LG17	0.999791751	110.711	151	99	1.12	13.03	96.97
LG18	0.999806121	112.865	147	110	1.03	5.41	98.18
LG19	0.999720432	96.247	103	91	1.06	8.92	97.8
LG20	0.999714443	120.244	114	91	1.32	12.51	94.51
Total		2337.882	2965	2445	0.956	18.44	98.2

Max Gap: The largest Gap in the linkage group. The smaller the maximum Gap is, the more uniform the map is. Gap < 5 cM: the proportion of bin markers whose Gap is less than 5 cM to all bin markers. Spearman coefficient: The closer Spearman coefficient is to 1, the better the collinearity between the genetic graph and the physical graph.

### Quantitative trait loci identification

**FIGURE 3:**
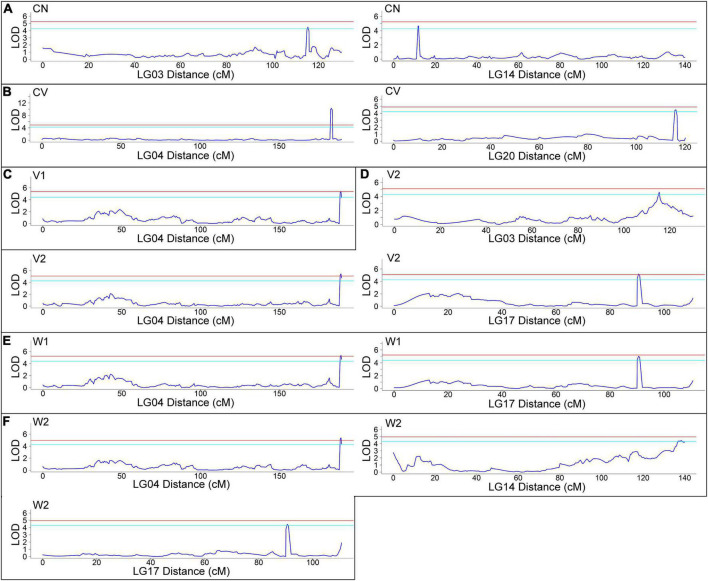
Quantitative trait loci (QTL) mapping of six traits related to fruit size from 102 F_2_ plants and two parents (AG and Hubbard). **(A)** Cell number (CN), **(B)** cell volume (CV), **(C)** fruit volume (V1), **(D)** fruit weight (W1), **(E)** fruit volume without seeds and placenta (V2) and **(F)** fruit weight without seeds and placenta (W2). The red line means a 0.01 (99% confidence) threshold level and the azure line means a 0.05 (95% confidence) threshold level for a candidate QTL.

Using joint analysis (LOD ≥ 4), based on the phenotyping data, 13 QTLs related to CN (2 QTLs), CV (2 QTLs), V1 (1 QTL), V2 (3 QTLs), W1 (2 QTLs) and W2 (3 QTLs) were identified (Figure 3). The information of each QTL, including LOD, map position, percentage of phenotypic variance explained (PVE) and threshold and LOD maximum values are presented in Table 2. Of note, several QTLs were found to be related to more than one trait, such as LG3 115.43 cM to 115.429 cM (related to both CN and V2) and LG4 188.58 cM to 189.561 cM (related to V1, V2, W1, and W2), confirming that there are intrinsic connections among these traits, as shown in Supplementary Table 3. These results indicated that fruit weight and volume, no matter whether include seeds and placenta, are closely correlated each other, and both CN and CV make important contributions of the large fruit of AG. Thus, genes located in these QTLs are worthy of our attention. Detailed gene information located in these QTLs is listed in Supplementary Table 6.

**TABLE 2:** Genetic mapping and quantitative trait locus analysis of 4 seed-related traits in 100 F_2_ individuals.

Triait	LG ID	Start (cM)	End (cM)	Min (LOD)	Max (LOD)	Min (PVE%)	Max (PVE%)	Start (bp)	End (bp)
CN	LG03	115.43	115.429	4.483	4.483	16.09029329	18.32441785	7770003	7781760
CN	LG14	11.782	12	4.647	4.647	18.92850129	18.92890772	1298851	1343835
CV	LG04	182.7	183.678	9.743	10.246	35.590225	37.03511312	18536335	18644674
CV	LG20	115.83	116.322	4.377	4.913	15.01135274	18.35401604	8701462	8750248
V1	LG04	188.58	189.071	5.164	5.393	17.83358354	21.61142548	19414367	19495938
V2	LG03	115.43	115.429	4.605	4.605	17.06621369	18.77444921	7770003	7781760
V2	LG04	188.58	189.561	4.767	5.463	19.36394567	21.86076583	19414367	19814128
V2	LG17	90.09	91	4.485	5.194	18.33175041	20.90470268	7740462	7986582
W1	LG04	188.58	189.561	4.697	5.36	19.11051215	21.49636912	19414367	19814128
W1	LG17	90.09	91	4.485	4.999	18.33290159	20.20505156	7740462	7986582
W2	LG04	188.58	189.561	4.339	5.38	17.79393599	21.56842962	19414367	19814128
W2	LG14	137	138.495	4.375	4.425	17.16672181	18.10998139	13609485	14221370
W2	LG17	90.58	90.58	4.458	4.4584	17.40514744	18.23280147	7749751	7822436

### Differentially expressed genes in fruits between Hubbard and Atlantic Giant

**FIGURE 4:**
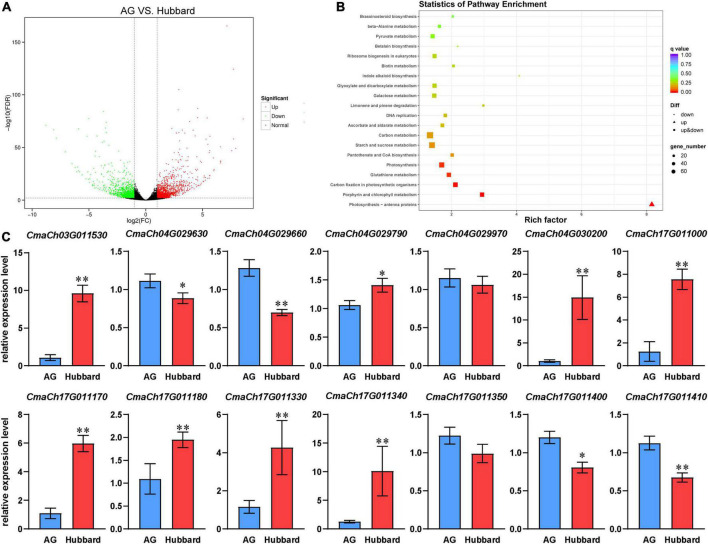
Differentially expressed genes (DEGs) of fruits at 20 days after anthesis between Hubbard and Atlantic Giant. **(A)** Volcano Plot of DEGs. **(B)** Top 20 significantly enriched Kyoto Encyclopedia of Genes and Genomes (KEGG) pathways. **(C)** Fourteen DEGs located in quantitative trait loci (QTL) regions and also related to more than one traits between Atlantic Giant (AG) and Hubbard samples were verified by qRT-PCR. Date are presented as means ± SD (*n* = 3, one-way ANOVA,*P < *0.05*,**P < *0.01*).

To further reveal gene expression regulation in pumpkin fruit size, we employed RNA-seq technology to analyze the gene expression profiles of fruits for Hubbard and AG at the fast growing stage (20 days after anthesis, DAA). RNA-seq produced 76.59 MB and 69.14 MB clean reads from Hubbard and AG (Q30 score ≥ 93%), and 95.07 and 95.17% of them could be mapped to the *C. maxima* genome, respectively. Between the Hubbard and AG groups, 2964 DEGs were identified (Figure 4A). Compared with Hubbard, the expression levels of 1332 genes were upregulated, and 1632 genes were downregulated in AG (Figure 4A). To elucidate the transcriptome differences between the two types of pumpkins, KEGG (Kyoto Encyclopedia of Genes and Genomes) analysis was carried out. We observed that some pathways associated with assimilate accumulation and fruit enlargement, such as carbohydrate metabolism and BR signaling, were significantly enriched (Figure 4B). Analysis of the RNA-Seq data for all genes located in QTL regions suggested that 32 genes were differentially expressed between AG and Hubbard, among which 14 genes were related to more than one trait (Supplementary Table 7). The expression differences of these transcripts were confirmed with qRT–PCR (Figure 4C).

### Candidate gene analysis

The SNP type among QTL genes was analyzed and only “missense_variant” was selected. Furthermore, 11 genes which have SNPs showed “Deleterious”calculated with PROVEAN were selected as candidate genes (Supplementary Table 8). In addition, insertion-deletion (inDel) mutations occurring in coding regions of QTL genes were also analyzed using PROVEAN, and two genes, *CmaCh04G029620* (annotated as the Noc2p family) and *CmaCh14G019420* (fatty acid hydroxylase superfamily), were also selected as candidate genes (Supplementary Table 9).

### Validation of candidate single nucleotide polymorphisms and inDels with Kompetitive Allele-Specific PCR markers

**FIGURE 5:**
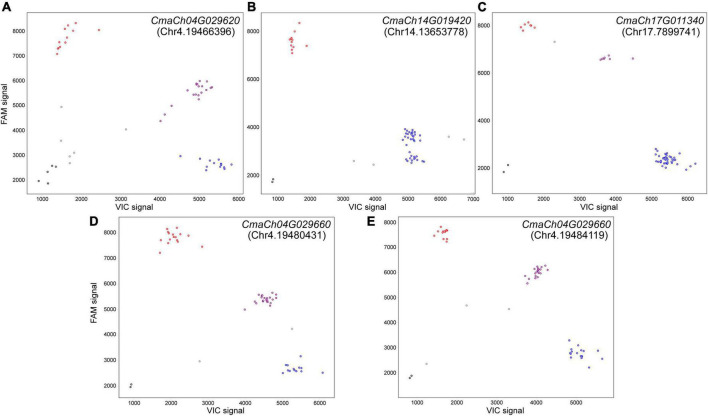
Analysis of the Kompetitive Allele Specific PCR (KASP) Results for genotyping. Scatter plots show the selected KASP assays clustering of varieties on the X-(VIC) and Y-(FAM) axes. The purple dot represents heterozygous genotype and blue and red dots represent the homozygous genotype of two parents (Atlantic Giant and Hubbard), 40 F_2_ individual plants and six cultivars showing extreme fruit sizes. The black dots represent the genotype is dropped out and gray dots represent the NTC (non-template control). The positions of these insertion-deletions (inDels) and Single Nucleotide Polymorphism (SNPs) were presented in parentheses. Marker for **(A)** inDel in *CmaCh04G029620*, **(C)** SNP in *CmaCh17G011340* and **(D,E)** SNP in *CmaCh04G029660* showing three distinct clusters. **(B)** Marker for inDel in *CmaCh14G019420* showing two distinct clusters.

Phytohormones have been reported closely associated with plant organ size. Therefore, two phytohormone-related genes, *CmaCh17G011340* (annotated as YUCCA) and *CmaCh04G029660* (annotated as leucine-rich repeat protein kinase family protein, LRR-RLK), were selected from candidate SNPs genes. Further analysis indicated that the “missense_variant” SNPs occurred in the CzcO domain of *CmaCh17G011340* and the PKc_like superfamily domain of *CmaCh04G029660*. KASP analysis was designed for the selected SNPs and inDels mentioned above. Finally, three SNPs and 2 inDels were selected to develop KASP marker and employed to screen two parents, 40 F_2_ individual plants and six cultivars showing extreme phenotypes to confirm their polymorphisms (Supplementary Table 10). The obtained results showed that four out of five markers distinguished the bulks, parents and six selected cultivars successfully (Figure 5), suggesting that these KASP markers were applicable for MAS of pumpkin fruit size breeding. These results further confirm that *CmaCh04G029660*, *CmaCh17G011340* and *CmaCh04G029620* are important candidate genes for fruit size control of *C. maxima*.

### Phylogenetic analysis and expression pattern of *YUCCA* and *LRR-RLK* family genes in *Cucurbita maxima*

**FIGURE 6:**
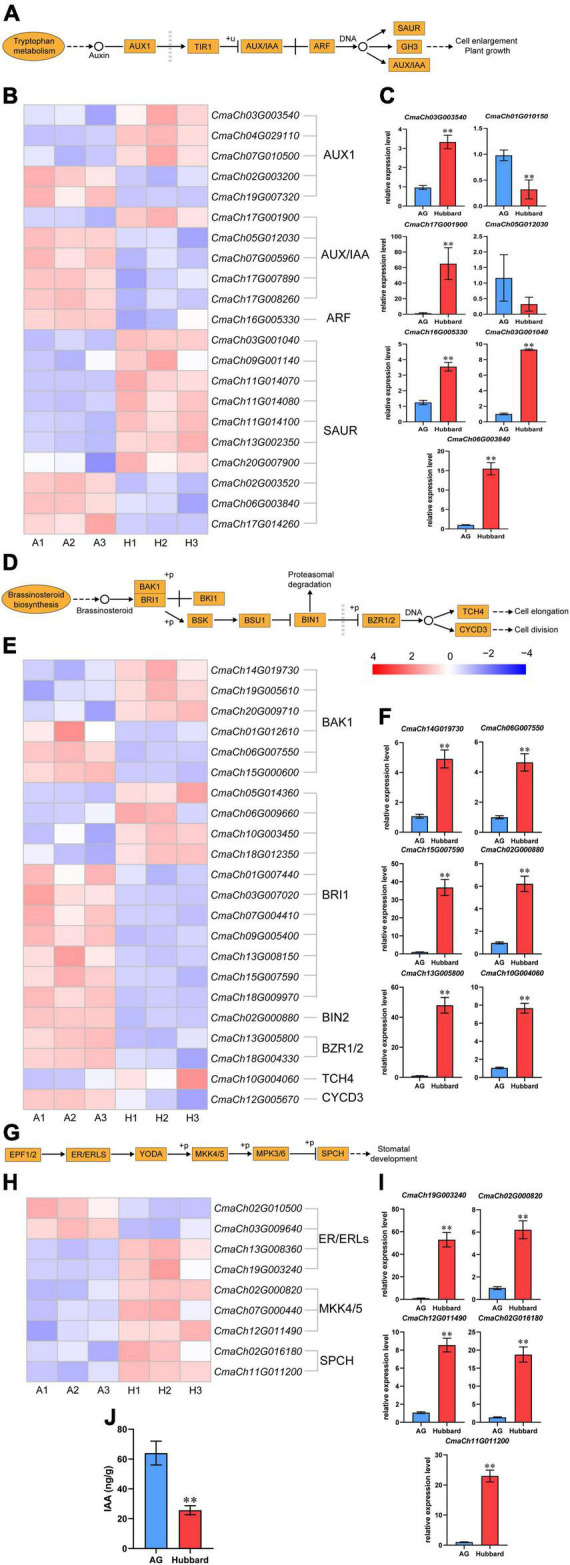
Visualization and validation of transcript expression in three pathways. The pathways were related to indole-3-acetic acid (IAA) **(A)**, brassinosteroid (BR) **(D)** in plant hormone signal transduction and ERECTA (ER) **(G)** in MAPK signaling pathway-plant. **(B,E,H)** The heatmap was plotted using Fragments Per Kilobase Million (FPKM) from RNA-seq data. **(C,F,I)** The expression levels of some differentially expressed genes (DEGs) involved in three pathway map between Atlantic Giant (AG) and Hubbard samples by qRT-PCR. **(J)** The level of IAA in AG and Hubbard. Date are presented as means ± SD (*n* = 3, one-way ANOVA, *P < 0.05, **P < 0.01).

Because little is known about the relationship between most candidate genes and organ size, only two phytohormone related genes *CmaCh17G011340* and *CmaCh04G029660* were further analyzed. Thirty-nine putative YUCCA from *C. maxima* (14), Arabidopsis (11) and rice (14) and 746 putative LRR-RLK family proteins from *C. maxima* (296), Arabidopsis (224) and maize (226) were collected for phylogenetic analysis (Supplementary Figures 4 and 6). Consistent with previous reports, the obtained results indicate that plant YUCCAs (Schlaich, 2007) are divided into three groups and LRR-RLK (Hosseini et al., 2020) into 23 groups. According to the function of genes closely related in the phylogenetic trees, *CmaCh17G011340* is considered a typical *YUCCA* family gene, whose function is to catalyze the rate-limiting step in the indole-3-acetic acid (IAA) synthesis (Schlaich, 2007). *CmaCh04G029660* belongs to the subfamily VI-1 group of *LRR-RLKs*. LRR-RLKs are the largest family of transmembrane receptors in plants and have been reported to be associated with various processes, including those associated with the BR and ERECTA (ER) signaling pathways (He et al., 2020). The functional domains of the two family genes and locations of SNPs are shown in Supplementary Figures 5 and 7. The evaluated impact of SNPs on the biological function of certain proteins (Supplementary Table 8) indicates that the isoforms of *CmaCh17G011340* in AG and *CmaCh04G029660* in Hubbard have higher activities. According to these results, it can be deduced that *CmaCh17G011340* positively regulates pumpkin fruit size, while *CmaCh04G029660* negatively regulates pumpkin fruit size. To further investigate the functions of the two candidate genes, the expression patterns of cascade genes in IAA, BR and ER signaling pathways were investigated. As shown in Figures 6A–H, the expression level differences of some important downstream genes, such as AUX/IAA, ARF and SAUR of the IAA signaling pathway, BIN2, BNR1/2 and TCH4 of the BR signaling pathway and MKK4/5 and SPCH of the ER pathway, between the two types of pumpkin were found, some of which were further confirmed by qRT–PCR. In addition, the IAA levels at the fruit fast-growing stage were also higher in AG fruits than in Hubbard fruits (Figure 6K). These results indicate that the SNPs occurring within *CmaCh17G011340* and *CmaCh04G029660* may improve fruit enlargement by altering the IAA, BR and ER signaling pathways. Of note, the mRNA abundance of *CmaCh04G029660* was higher and that of *CmaCh17G011340* was lower in AG than in Hubbard (Figure 4C), indicating that the alterations in the IAA, BR and ER pathways were not due to changes in gene expression levels. Therefore, the SNP in the coding region of these two genes between the two pumpkins may be a key reason to alter protein function and further affect the biosynthesis of IAA and the downstream signaling pathways of BR and ER. As shown in Supplementary Table 5, we also noticed SNPs in the promoter regions of the two candidate genes. Whether these promoter SNPs are reasons for the expression difference of the two genes between the two pumpkins needs to be further studied.

## Discussion

In this study, all 102 F_2_ plants were used for genome-wide resequencing. Bulked segregation analysis (BSA) is another choice that has been widely used for constructing genetic linkage map and identifying QTL due to its advantages of cost-effectiveness and relatively low time consumption (Tang et al., 2017). However, because the space required for giant pumpkin growth is vast, the population size of F_2_ and F_2:3_ was limited in our study. The skewed normal distribution (skewed toward the small fruit parent) of all tested traits further led to the deficiency of F_2_ lines for big-fruit mixed poll construction (Figure 1B). Thus, all available F_2_ plants were used for SNP calling and QTL detection in this study. Furthermore, with the application of next-generation DNA sequencing (NGS), an increasing number of reduced representation sequencing methods have been applied in recent molecular research, such as genotyping by sequencing (GBS) and restriction-site associated DNA sequencing (RAD-seq; Meger et al., 2019). In comparison with these methods, a whole-genome resequencing (WGRS)-based approach could identify more polymorphism SNPs randomly distributed throughout the genome, which is beneficial for decreasing the candidate regions (Zhao et al., 2020). Therefore, WGRS was performed for all F_2_ lines in this study. In addition, bin mapping has been proven to be an efficient method for generating high-density genetic maps for identifying QTLs in many plant species (Wang et al., 2019). Finally, with the application of the WGRS-based bin-map strategy, we identified more SNP and bin markers than previous works of *C. maxima* genetic linkage map construction (Zhang et al., 2015; Wang et al., 2020). Therefore, we suggest that the strategy employed in this study is an efficient approach for genetic linkage map construction and with a high density of SNP and bin markers. The constructed map should be a powerful tool for the fine mapping of QTLs and marker-assisted breeding of important traits of *C. maxima*.

Perhaps due to their economic value, substantial QTLs and candidate genes associated with fruit size of cucumber, melon and watermelon have been identified (Pan et al., 2020). Although fruit weight variation among pumpkins is more fascinating, only few researches have been performed to elucidate the genetic mechanism of fruit weight variation in *Cucurbita* crops (Pan et al., 2020). Kaźmińska et al. (2020) used recombinant inbred lines (RILs) to identify QTLs related to the fruit weight. In this study, the fruit weight of one parent was approximately 5-fold higher than that of another parent. Six QTLs for fruit size were detected, and the major-effect QTL explaining 32 and 41% of the phenotypic variation in the two experiments was located on chromosome 4 (*p* ≤ 0.01). The remaining fruit weight QTLs were located on chromosomes 2, 10, 14 and 17. In our study, we also detected fruit size-associated QTLs on chromosomes 4, 14 and 17. However, most genomic positions of these QTLs were different between the two experiments. The only overlapping fragment of fruit weight-associated QTLs between the two experiments was from 13609485 to 13764003 on chromosome 14. Twenty-two genes (*CmaCh14G019320* – *CmaCh14G019560*) were identified in this region (Supplementary Table 11). Among these genes, we noted that *CmaCh14G019450*, encoding a WD40 repeat-like superfamily protein, also showed a “Deleterious” prediction in PROVEAN calculation. Yang et al. (2018) also reported that *LITTLELEAF* encodes a WD40 repeat protein related to organ size in cucumber. Thus *CmaCh14G019450* should be another important candidate gene and worthy further investigation.

IAA is the most common auxin and plays various roles in plant development and response to abiotic and biotic stress. In higher plants, the tryptophan-dependent pathway is the predominant IAA biosynthesis pathway, among which TRYPTOPHAN AMINOTRANSFERASE OF ARABIDOPSIS (TAA) and flavin monooxygenase (YUCCA) are two key catalytic enzymes (Zhao, 2014). After biosynthesis, the IAA function is achieved through several processes, including IAA transport, metabolism, and signal transduction (Devoghalaere et al., 2012). These processes were coordinated by a series of regulators, such as gretchen hagen 3 (GH3) family protein (Staswick et al., 2002; converting active IAA to inactive IAA),auxin-resistant 1/like auxin-resistant 1 (AUX1/LAX1) and PINformed 1 (PIN1; transports auxin between extracellular and intracellular regions), small auxin-up RNA (SAUR), auxin response factors (ARFs) and transport inhibitor response1/auxin signaling F-BOX protein (TIR1/AFB; Staswick et al., 2002; Paponov et al., 2008; Wabnik et al., 2010; Zažímalová et al., 2010; Lavy and Estelle, 2016; Leyser, 2018). The size of various plant organs has been reported to be affected by the IAA level, including fruits, seeds and roots (Chhun et al., 2007; Figueiredo and Köhler, 2018; Bu et al., 2020). IAA significantly increases organ size by promoting cell division and enlargement (Ljung et al., 2001). According to Supplementary Figures 4 and 8, the expression of two homologs of *CmaCh17G011340* was not detected in fruits of either variety, further indicating the important role of *CmaCh17G011340* during fruit enlargement.

Receptor-like kinases represent the largest family of cell surface receptors in plants, among which LRR-RLKs are one of the largest RLK subgroups (Schlaich, 2007). LRR-RLKs characteristically contain an intracellular kinase domain, a transmembrane domain and an extracellular LRR, whose function is to transduce signal in and out of cells (He et al., 2020). According to different phylogenetic analysis methods, the LRR-RLK subfamily could be divided into different number of clusters (Liu et al., 2016, 2017; Sun et al., 2018). Sakamoto et al. (2012) proposed that the LRR-RLKII subfamily contains three well-supported clades, named SERK (somatic embryogenesis receptor kinase), NIK (NSP-interacting kinase) and LRRIIc (unknown function). However, based on the combined extracellular and intracellular sequence matrix, Hosseini et al. (2020) redefined LRR-RLKIIs into five subgroups named LRR-RLKII 1–5. In the last decade, several LRR-RLK-associated signaling pathways have been reported in Arabidopsis and other plants (Zhang et al., 2012; He et al., 2020). A receptor complex containing two LRR-receptor kinases, BRASSINOSTEROID INSENSITIVE1 (BRI1, belonging to subfamily Xb-1) and BRI1ASSOCIATED KINASE-1 (BAK1), triggers phosphorylation of their intracellular domains (Hohmann et al., 2017). The phosphorylated kinases recruit and activate downstream transcription factors of BR signaling such as ASSINAZOLE-RESISTANT 1 (BZR1) and BRI1-EMS-SUPPRESSOR 1 (BES1), which eventually leads to vast changes in a variety of biological processes mediated by BR signaling (Wang et al., 2002; Yin et al., 2002). *ER*, another *LRR* family gene (subfamily XIIIb), functions with *ER-LIKE1* (*ERL1*) and *ERL2* synergistically to mediate multiple growth processes, including organ size, inflorescence architecture, petiole length, ovule development, and stomatal patterning (Shpak, 2004, 2013; Torii, 1996; Pillitteri et al., 2007). *ZmRLK7*, an *LRR-RLK* of *Zea mays*, negatively regulates plant organ size by influencing the SERKs, ER cascade and BR cascade (He et al., 2020). In our experiment, *CmaCh04G029660* belongs to subfamily VI-1; unfortunately, little research has been conducted in this group. According to our results, AG fruit has a less active allele of this LRR-RLK than Hubbard fruit, and the expression of abundant downstream genes of the BR and ER pathways was different between the two types of pumpkins, indicating that *CmaCh04G029660* may also act as a negative regulator of pumpkin fruit by affecting BRs and ER signaling pathways. If this is true, the exact mechanism of regulation is worthy of further study. We cannot rule out other pathways of the regulation conducted by *CmaCh04G029660*. In addition, several other *LRR* genes, which have high homology with *CmaCh04G029660*, were also expressed in AG and Hubbard fruits (Supplementary Figures 6 and 9). The role of these *LRRs* in fruit enlargement needs to be further investigated.

In this study, a genetic map with an average physical interval of 154 kb per marker was constructed using GWRS and Bin marker technology. Thirteen fruit size-related QTLs were further identified on chromosomes 3, 4, 14, and 17 of *C. maxima*. Additional RNA-Seq data and impact analysis of SNP and inDel suggested several important candidate genes, such as *CmaCh04G029620*, *CmaCh04G029660*, *CmaCh14G019420, CmaCh14G019450* and *CmaCh17G011340*. In addition, four KASP markers that were closely linked to fruit size were developed to confirm the SNP and inDel mutations and provide an MAS tool for *C. maxima* breeding. These data can be used to understand the molecular mechanisms underlying the formation of the world’s largest fruit and provide candidate genes for fruit size-related breeding of *C. maxima*. Furthermore, two SNPs were identified in the key domains of *CmaCh17G011340* and *CmaCh04G029660*, and significant differences in the IAA level and RNA abundance of several genes in the IAA, BR and ER signaling pathways were found between large and small pumpkins, indicating that IAA, BR and ER may play important roles in the AG fruit enlargement. In addition, compared with Hubbard, AG has several other morphological and physiological characteristics facilitating its fast fruit growth, such as larger leaves, higher leaf stachyose level, higher net photosynthetic rate, and larger peduncle vascular cross area (Pan et al., 2021). Whether IAA, BR and ER play roles in these biological processes is worth further study. Functional verification by gene editing of two candidate genes is being carried out in our lab.

## Data availability statement

The original contributions presented in the study are publicly available. This data can be found here: NCBI, PRJNA797700, and PRJNA797701.

## Author contributions

MM designed the experiments and coordinated the project. LP, MW, YY, and CC performed the experiments, analyzed the data, and drew conclusions based on the results. MM wrote the manuscript. HD, ZZ, and BH helped to perform the analysis with the constructive discussions. All authors read and approved the final manuscript.

## Conflict of interest

The authors declare that the research was conducted in the absence of any commercial or financial relationships that could be construed as a potential conflict of interest.

## Publisher’s note

All claims expressed in this article are solely those of the authors and do not necessarily represent those of their affiliated organizations, or those of the publisher, the editors and the reviewers. Any product that may be evaluated in this article, or claim that may be made by its manufacturer, is not guaranteed or endorsed by the publisher.

## Footnotes

1
http://www.tiangen.com/en/?productShow/t1/4/id/10.html

2
http://www.takarabiomed.com.cn

3
www.genomics.cn

4
http://www.novogene.com/

5
http://cucurbitgenomics.org/organism/8

6
https://broadinstitute.github.io/picard/

7
http://www.kegg.jp

8
http://www.arabidopsis.org/
