# Supplementary material for: Whole-genome resequencing identified QTLs, candidate genes and Kompetitive Allele-Specific PCR markers associated with the large fruit of Atlantic Giant (Cucurbita maxima)
Source: Front Plant Sci. 2022 Jul 22;13:942004. doi: 10.3389/fpls.2022.942004 (PMC9354748; doi:10.3389/fpls.2022.942004)
Supplement: Supplementary file 1 [file Image_1.PDF]

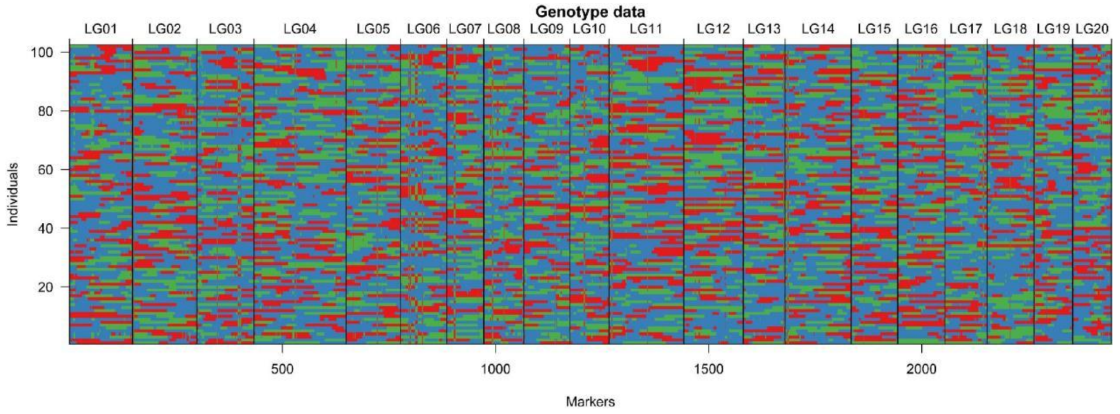

Supplementary Figure 1 The recombination bin-map of the illustrated genotype in the population. The red segment shows chromosomes from the paternal genome, the green segment from the maternal genome, and the blue heterozygous segment indicates the size of the chromosome on the horizontal axis and the number of the offspring on the vertical axis.

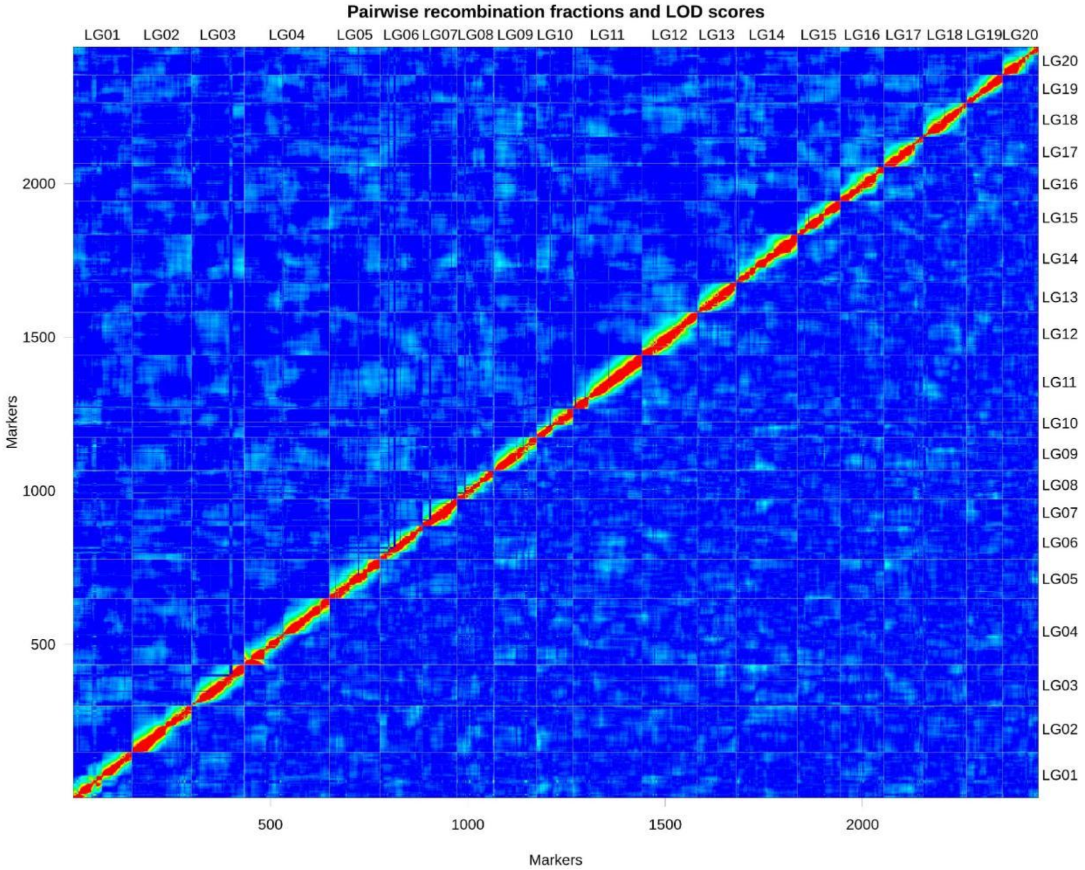

Supplementary Figure 2 The heat maps of pairwise recombination fractions and Logarithm of

the Odds (LOD) scores of genetic map.

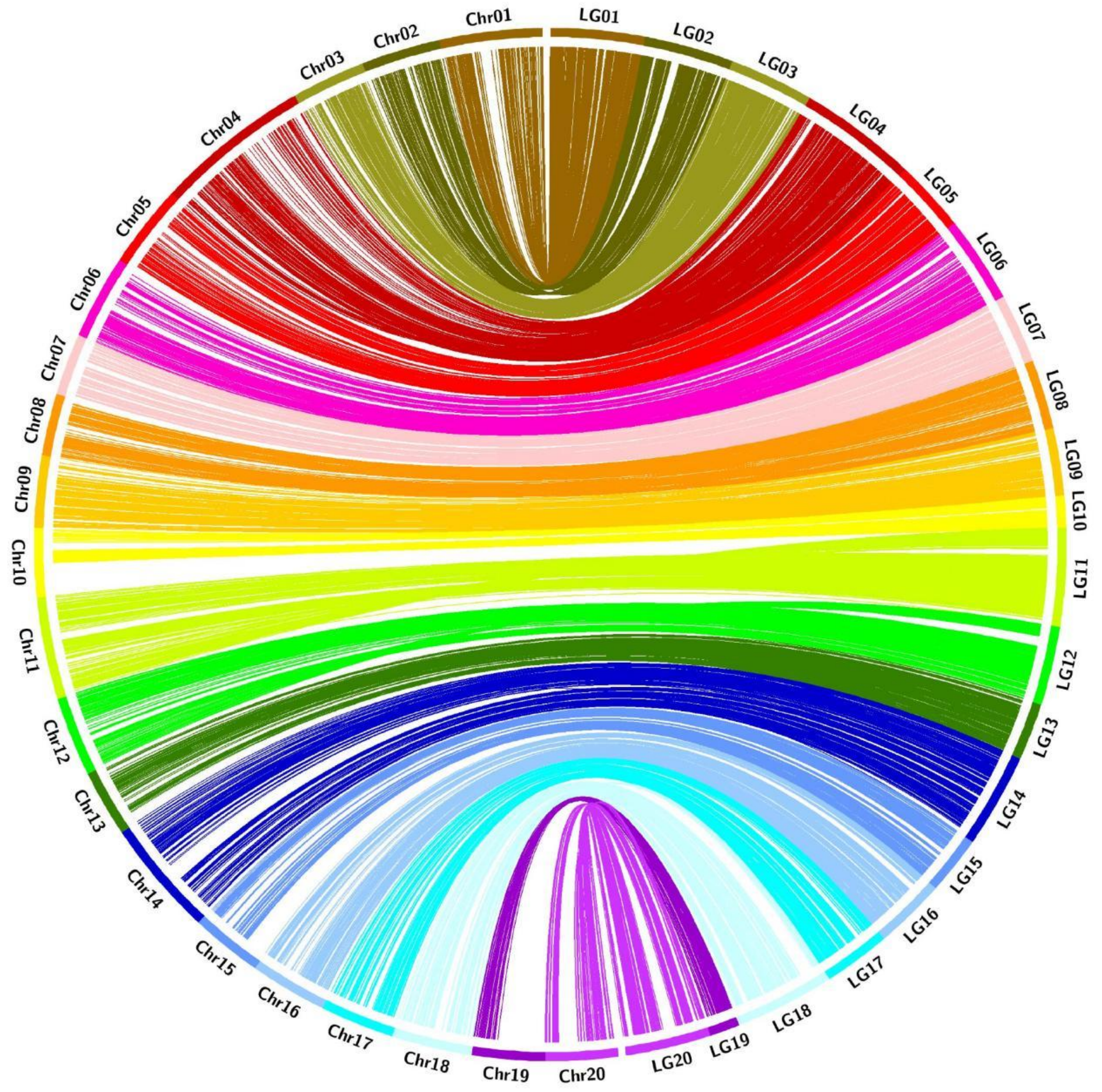

Supplementary Figure 3 The synteny analysis of genetic maps and genomes.

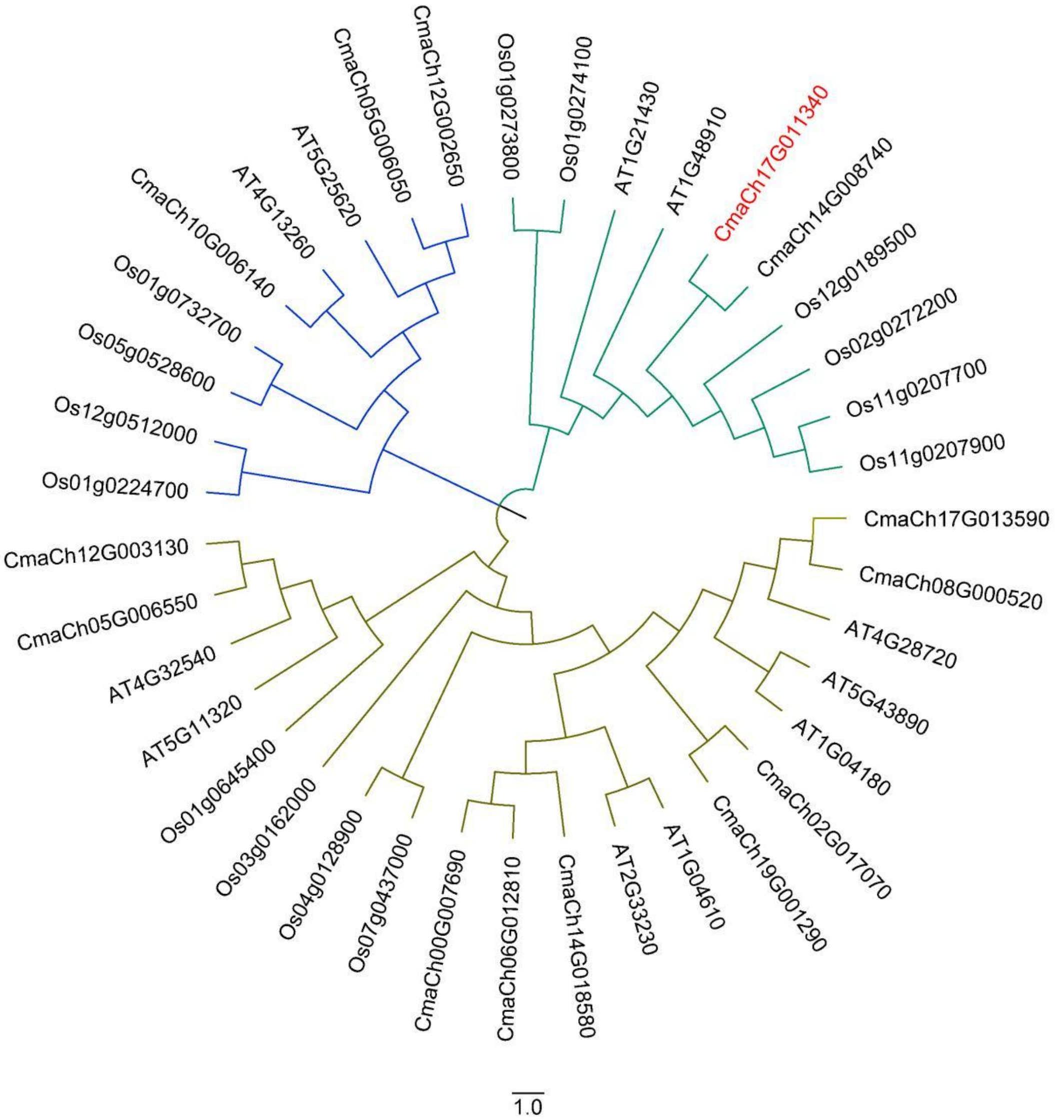

Supplementary Figure 4 The phylogenetic tree for YUCCA proteins identified in *Cucurbita maxima*, Arabidopsis and rice. A total of 39 YUCCA proteins. were classified into 3 subfamilies and distinguished by different colors. Candidate gene (*CmaCh17G011340*) was marked in red.

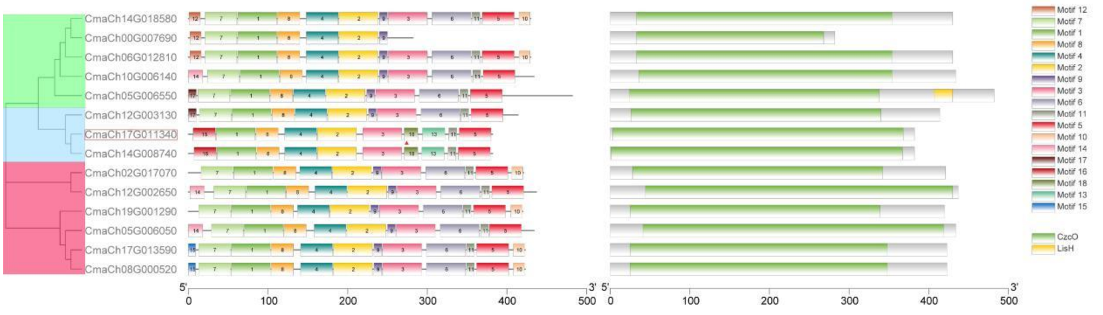

Supplementary Figure 5 Phylogenetic tree, motifs and domains of *Cucurbita maxima* YUCCA

proteins. Left: Phylogenetic tree of *Cucurbita maxima* YUCCA proteins. Candidate gene (*CmaCh17G011340*) was marked in red. Middle: Motifs were identified using MEME online tool and illustrated in different colors. The red triangle represent the position of the Single Nucleotide Polymorphism (SNP) Right: Domains were also identified using NCBI CDD online tool and illustrated in different colors.

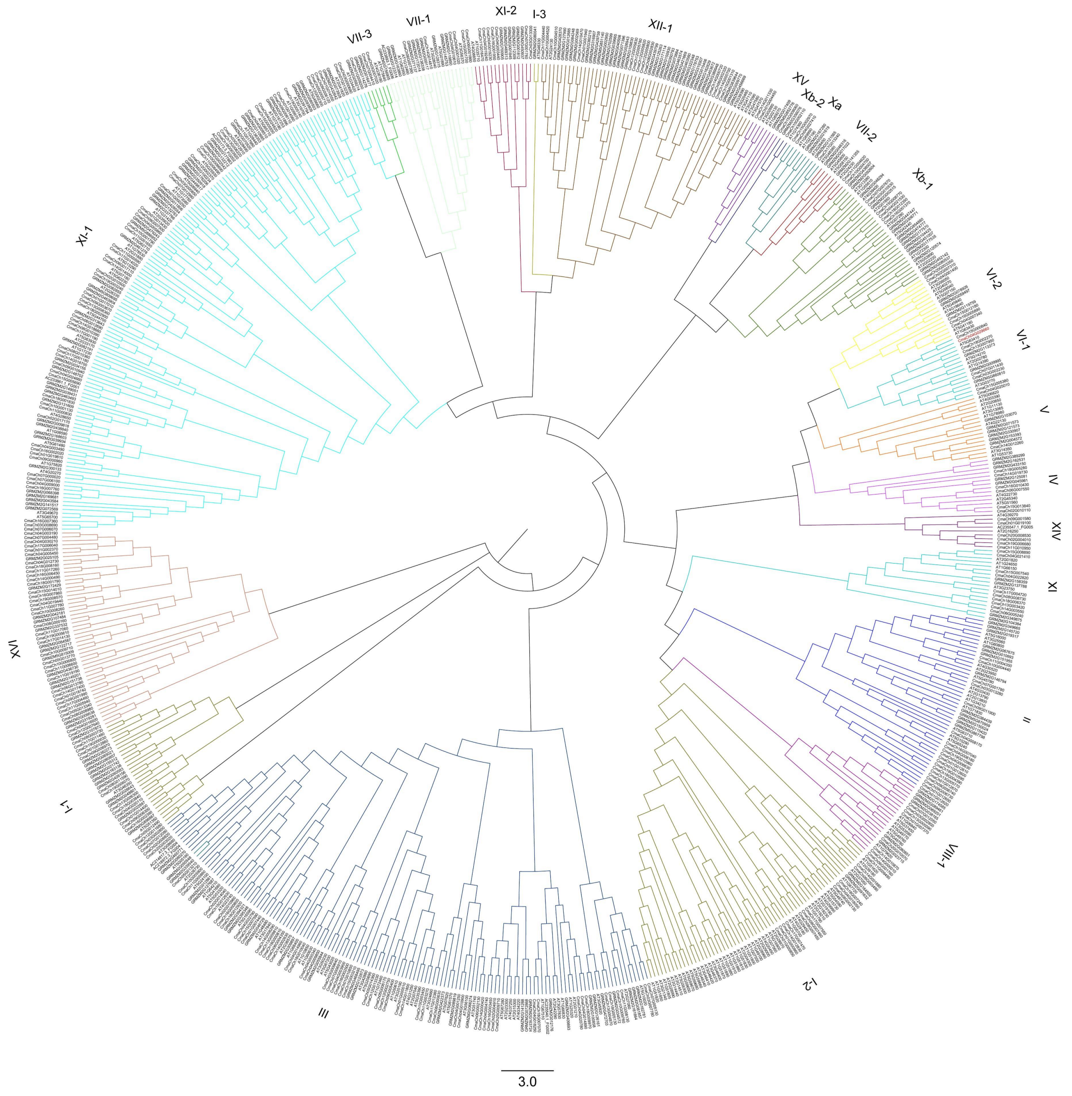

Supplementary Figure 6 The phylogenetic tree for LRR-RLK proteins identified in *Cucurbita maxima*, Arabidopsis and maize. A total of 746 LRR-RLK proteins were classified into 23 subfamilies and distinguished by different colors. Candidate gene (*CmaCh04G029660*) was marked in red.

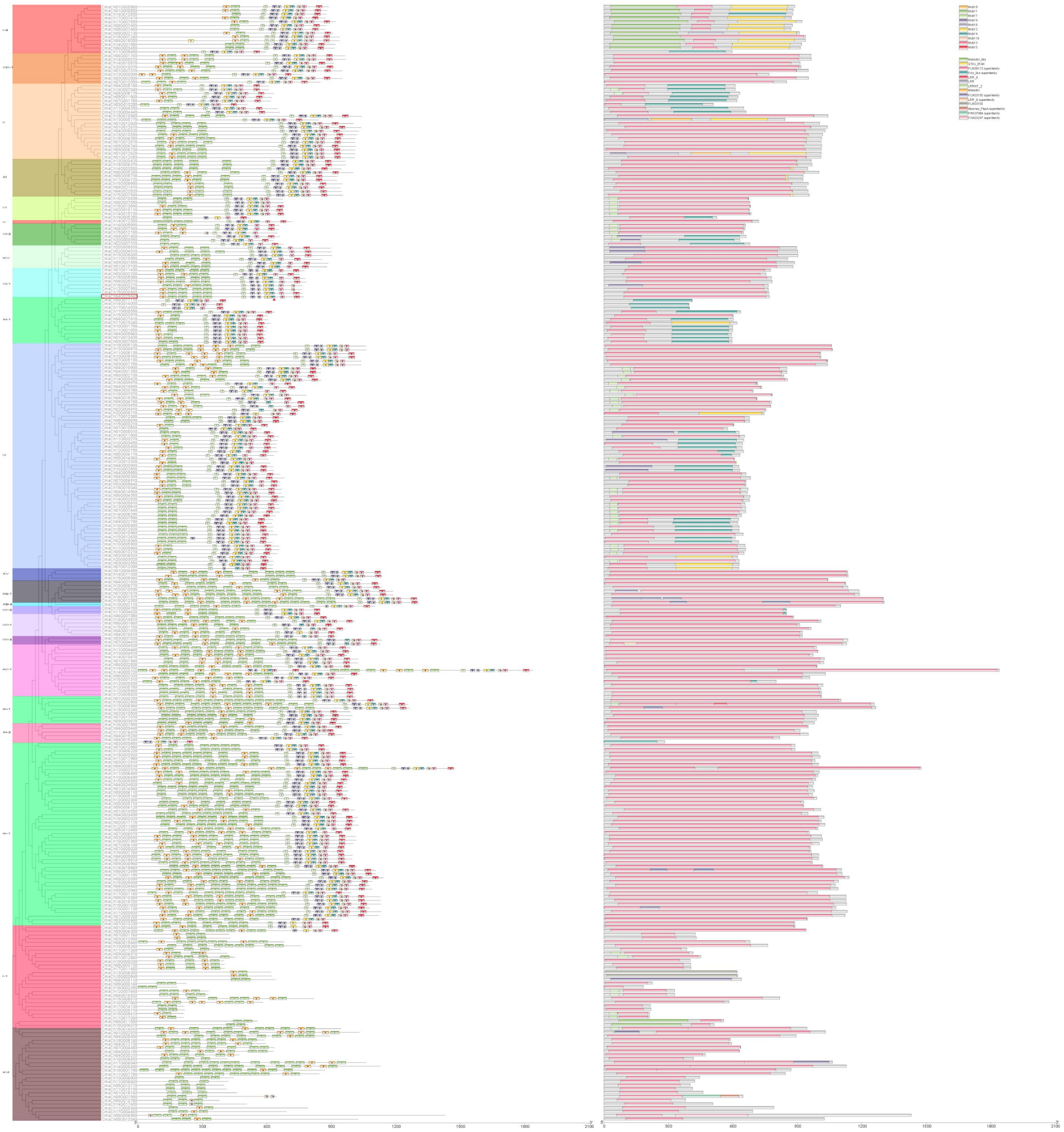

Supplementary Figure 7 Phylogenetic tree, motifs and domains of *Cucurbita maxima* LRR-RLK proteins and alignment of sequences. Left: Phylogenetic tree of *Cucurbita maxima* LRR-RLK proteins. Candidate gene (*CmaCh04G029660*) was marked in red.. Middle: Motifs were identified using MEME online tool and illustrated in different colors. The red triangle represent the position of the Single Nucleotide Polymorphism (SNP). Right: Domains were also identified using NCBI CDD online tool and illustrated in different colors.

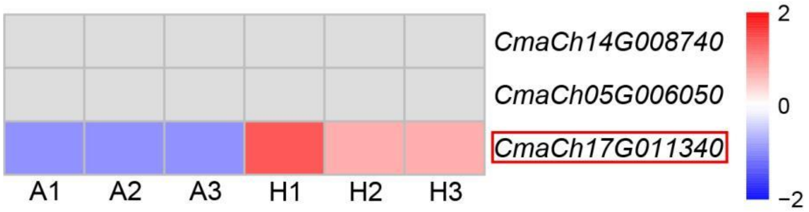

Supplementary Figure 8 The heatmap indicating expression levels of *CmaCh17G011340* and 2 genes clustered near *CmaCh17G011340* between "Atlantic Giant" and "Hubbard".

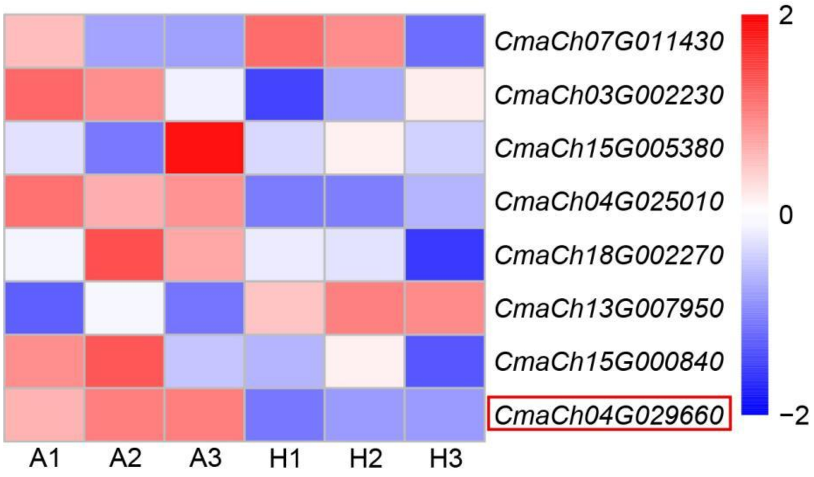

Supplementary Figure 9 The heatmap indicating expression levels of *CmaCh04G029660* and 7 genes clustered near *CmaCh04G029660* between "Atlantic Giant" and "Hubbard".
